# Liver stiffness measurements predict Sinusoidal Obstructive Syndrome after hematopoietic stem cell transplantation

**DOI:** 10.1038/s41409-024-02288-1

**Published:** 2024-04-24

**Authors:** Yana Davidov, Noga Shem-Tov, Ronit Yerushalmi, Tammy Hod, Ziv Ben-Ari, Arnon Nagler, Avichai Shimoni, Ivetta Danylesko

**Affiliations:** 1 Liver Diseases Center, Sheba Medical Center, Tel-Hashomer, Israel; 2 Faculty of Medicine, Tel-Aviv University, Tel-Aviv, Israel; 3 The Division of Hematology and Bone Marrow Transplantation, Sheba Medical Center, Tel-Hashomer, Israel; 4 Renal Transplant Center and Nephrology department, Sheba Medical Center, Tel-Hashomer, Israel

**Keywords:** Haematopoietic stem cells, Disease prevention

## Abstract

Sinusoidal Obstructive Syndrome (SOS) is a life-threatening complication after hematopoietic stem-cell transplantation (HSCT), characterized by post-sinusoidal portal hypertension. FibroScan is used to assess portal hypertension non-invasively. We assessed transient elastography (TE) applicability in diagnosing SOS. The study included 27 adult patients, 11 underwent TE for high SOS risk pre-HSCT, 17 underwent TE post-HSCT due to bilirubin ≥2 mg/dl with no definite diagnosis of SOS. The first group had median Liver Stiffness Measurement (LSM) of 7.4 kPa (range, 3.3–22.5). Based on LSM results, conditioning regimen was modified for six patients and two of them developed SOS. Only one patient who did not have protocol adjustment experienced SOS. No patient with LSM < 7 kPa developed SOS. The second group had median LSM of 7.7 kPa (4.4–31.5). Median LSM after HSCT was significantly higher in patients who subsequently developed established SOS (*n* = 10) compared to patients who did not (*n* = 8), with values of 10.7 kPa (5.6–31.5) and 5.9 kPa (4.4–13.8), respectively (*p* = 0.02). An LSM cut-off of 7.5 kPa had a sensitivity and specificity of 75 and 80% for diagnosing SOS. In conclusion, pre-HSCT LSM can help adjustment of conditioning regimen in patients with high-risk for SOS. Post-HSCT LSM can help in early diagnosis of SOS.

## Introduction

Sinusoidal Obstructive Syndrome (SOS) is a rare but potentially serious complication that can occur after hematopoietic stem cell transplantation (HSCT). On average, the incidence of SOS in adults is 10% [1]. In severe cases, the mortality rate can be as high as 80% [2]. The pathogenesis of SOS after HSCT involves the injury of the sinusoidal endothelial cells of the liver, which leads to loss of wall integrity, endothelial cell detachment, downstream embolization of the centrilobular vein, and sinusoidal obstruction [2, 3]. These events result in outflow obstruction, causing hepatic congestion and the development of post-sinusoidal portal hypertension [2]. The clinical presentation of SOS is the consequence of portal hypertension, and the severity can vary. Typical clinical features of SOS in adults are hyperbilirubinemia, painful hepatomegaly, ascites, and weight gain [2]. In severe cases, SOS can progress to liver failure and multi-organ dysfunction [2, 4].

There are several risk factors for SOS [5]. A number of widely recognized risk factors for SOS onset have been established related to patients and disease characteristics: pre-existing liver diseases, obesity, previous abdominal/hepatic radiation, previous treatment with Gemtuzumab ozogamicin, Inotuzumab ozogamicin, multiple lines of previous chemotherapy, previous HSCT, baseline elevated bilirubin, transaminase, ferritin, iron overload, older age for adults patients, poor performance status, advanced disease states and HLA-mismatched donor. In addition, the likelihood of developing SOS varies depending on the intensity of treatment and the drugs administered. Using high doses of Busulfan (BU) or total body irradiation (TBI)-based myeloablative conditioning (MAC) can increase the risk, while reduced intensity conditioning (RIC) can reduce it. Moreover, a high dose of TBI, a combination of BU, Cyclophosphamide (CY), Dual alkylating agent conditioning (DAC), and methotrexate (MTX) -based Graft versus host disease (GVHD) prophylaxis can also increase the risk of SOS [5].

The diagnosis of SOS is based on European Society for Blood and Marrow Transplantation (EBMT) criteria [1, 4, 5] which include clinical criteria and imaging findings. The mandatory factor in EBMT criteria is the elevation of bilirubin above 2 mg/dl [4]. The EBMT criteria’s specificity is relatively low, as other conditions can imitate the symptoms of SOS. The differential diagnosis of bilirubin elevation and fluid retention in patients after HSCT includes reactivation of viral disease, sepsis, drug-induced liver injury other than SOS, graft versus host disease (GVHD), hepatic vein or portal vein thrombosis [2]. Imaging techniques can help detect SOS by showing signs of liver congestion, portal hypertension, and parenchymal damage [6]. However, imaging findings are often nonspecific and may overlap with other liver disorders. Therefore, imaging techniques are usually used as an adjunct to clinical and laboratory criteria for diagnosing SOS [2].

Liver stiffness measurement (LSM) is a non-invasive diagnostic tool to assess liver fibrosis and portal hypertension [7, 8]. While LSM helps in diagnosing and monitoring of liver fibrosis, it has limited use in diagnosing SOS. In recent years, there have been several reports of SOS assessment using LSM techniques such as transient elastography (TE), point shear wave with acoustic radiation force impulse (ARFI), and two-dimensional real-time shear wave (2D-SWE) [2, 6, 9–15]. Debureaux and colleagues prospectively assessed LSM before and after allo-HSCT [9]. Baseline LSM did not predict the development of SOS, but it did significantly increase in patients who did develop it [9].

Our study aimed to assess the applicability of LSM in guiding the selection of the conditioning regimen before transplantation and the diagnostic accuracy of LSM in identifying SOS after allogeneic HSCT (allo-HSCT).

## Material and methods

### Study population

In this study, we performed TE using FibroScan in adults (>18 years old) before or after allo-HSCT at Sheba Medical Center between January 2021 and June 2023. Patient demographics, clinical and laboratory characteristics were recorded before and after allo-HSCT. Liver stiffness measurement was assessed for all patients with FibroScan (Echosens, Paris, France). TE was performed after fasting over 4 h. The reliability criterion was at least ten measurements with a ratio of the interquartile range (IQR) of liver stiffness to the median (IQR/M) ≤ 30% [8].

The main objectives in the first group were to assess the correlation of LSM before HSCT in high-risk patients for the development of SOS and the impact of modification of the planned conditioning regimen in reducing the risk for SOS.

To rule out the possibility of having clinically significant liver disease, we chose a low threshold of 7 kPa [9, 16]. Patients with an LSM of 7 kPa or less remained with standard treatment protocol despite elevated liver enzymes before allo-HSCT. However, for patients whose LSM was higher than 7, we adjusted their treatment to a low-toxicity option.

In the second group we investigated the diagnostic ability of LSM to differentiate SOS from other causes of liver injury after HSCT. We also aimed to determine the cut-off LSM value for the diagnosis of SOS. The second group consisted of patients with an elevation of bilirubin ≥2 mg/dl after the allo-HSCT within 21 days after transplantation with or without fulfilling the EBMT clinical criteria for diagnosis of SOS. The diagnosis of SOS was confirmed when EBMT criteria were present [4, 5].

**Fig. 1:**
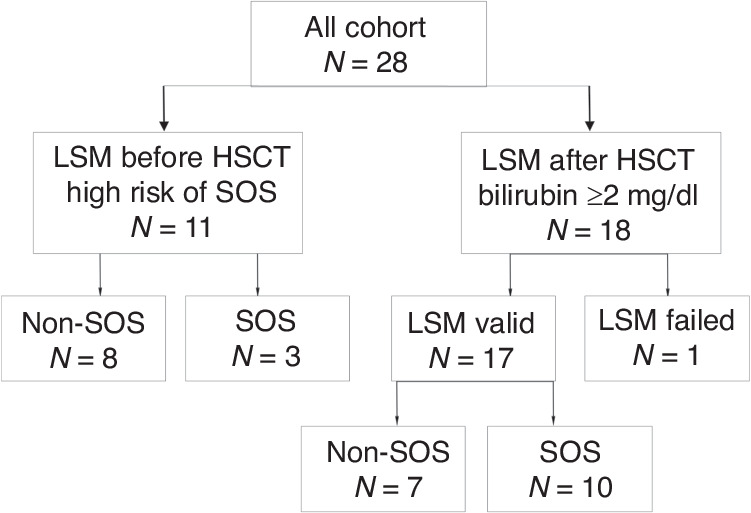
Flow chart of study cohort. Of 28 patients, 11 high-risk patients were assessed for the development of SOS before HSCT, and 18 of 28 patients were assessed after HSCT due to bilirubin levels above 2mg/dl.

The 1st group consists of 11 patients, and 2nd group of 17 patients (one patient was included in both groups) (Fig. 1).

#### Statistical analysis

The data for continuous variables is presented as a median with an interquartile range (IQR), while categorical variables are expressed as a count with a percentage. Patients in the 1st and 2nd groups were categorized based on their SOS diagnosis. Those who developed SOS were then compared to those who did not (the non-SOS group). To compare categorical variables, we used chi-squared analysis and Fisher exact test. We used the Student’s *t* test or the Mann–Whitney U test for continuous measurements, depending on their distribution. We performed a receiver operator characteristic (ROC) analysis to evaluate the accuracy of predicting SOS diagnosis. The cut-off values of LSM were determined using ROC analysis, and the best cut-off value was estimated using the Youden index. We considered *p* < 0.05 to be a statistically significant difference, and all tests were two-sided. We performed the statistical analysis using SPSS (IBM SPSS Statistics, version 25, IBM Corp., Armonk, NY, USA, 2016). Finally, we generated scatter plots of the analyzed data using GraphPad Prism version 10.0.0 for Windows (GraphPad Software, San Diego, USA).

## Results

### LSM before HSCT for assessment of high-risk patients for development SOS

Eleven patients at high risk of developing SOS were evaluated before HSCT. The clinical and demographic characteristics of these patients are presented in Table 1. The median age at the time of transplantation was 43 years (IQR, 37–60) and 45.5% of the patients were male.

**Table 1:** Baseline demographic and clinical characteristics of patients assessed before HSCT because of high-risk for development SOS.

*N*	DS	Age/Gender	Reason for baseline FS	*N* of factors	Donor	Planned protocol	Actual protocol	LSM kPa	Specific decisions per FS	Type of change	SOS after HSCT/severity
1	AML	22/F	-2^nd^ HSCT/ Multiple lines of Tx/Active disease/TBI	4	Haplo father	TBF	FLUTBI	10.1	No DAC	Composition change	No
2	sAML	40/F	-2^nd^ HSCT -Multiple lines of Tx -DAC	3	Haplo brother	TBF-RIC	TBF-RIC	7	No specific decision	No	Yes/Moderate
3	AML	23/M	Elevated liver enzymes	1	Sibling 10/10	FLUBU4	BUCY	4.9	Despite liver enzymes elevation - MAC BUCY	Intensification	No
4	sAML	42/F	-Alcohol abuse -Fatty liver	2	MUD 10/10	FLUBU4	FLU Treo 14	7.4	Treo instead of BU	Composition change	Yes/Mild
5	AML	60/F	-Age -2^nd^ HSCT -Multiple lines of Tx - Previous post CTX severe liver toxicity	4	Sibling 10/10	FLUBU2	FLU Treo 12	3.3	RTC instead RIC	Intensification	No
6	MF	37/M	-MF -Severe splenomegaly -Susp PHTN	3	MUD 10/10	TBF	TBF MUC	10.2	No specific decision	No	No
7	MF	51/M	-MF - Severe PHT d/t Portal vein thrombosis - Previous post CTX severe liver toxicity	3	Sibling 10/10	TBF	FLU Treo 12	22.5	Liver biopsy –N, No DAC, No BU, No MTX	Composition change	Very Severe/death
8	sAML	65/F	- Age - Fatty liver with NASH + liver cirrhosis - Past CTX for Ca of Breast	4	Haplo son	TBF	Baltimore RIC protocol mini dose FCYTBI 200 RAD	18	RIC/No DAC/Prophylactic Defibrotide	Composition change+ Deintensification	No
9	MF	46/M	-MF -Severe splenomegaly -Susp PHTN	3	Haplo son	TBF MAC	TBF RIC	11.6	RIC	Deintensification	No
10	MF	65/M	-MF -Severe splenomegaly -Susp PHTN -Age -Fatty liver disease	5	Sibling 10/10	TBF	TBF RIC	13.8	RIC	Deintensification	No
11	sAML	43/F	-Active disease -Primary hemochromatosis -Periodic Elevated liver enzymes	3	Sibling 10/10	FLUBU4 or FLU Treo12	FLU Treo12	5.1	RTC	No specific decision	No

*CTX* chemotherapy, *MF* myelofibrosis, *VV* Vidaza + Venetoclax, *DLBCL* Diffuse large B-cell lymphoma, *TBI* total Body irradiation, *FLU* fludarabin, *RIC* reduced intensity conditioning, *MUD* matched unrelated donor, *TBF* Thiothepa + Busulfan + Fludarabin, *BUCY* Busulfan + Cytoxan, *MUC* myelo-ablative conditioning, *FC* Fludarabin + Cytoxan.

Out of the 11 patients, 7 had AML, that was secondary or treatment-related in 4. The remaining four patients had myelofibrosis. Before undergoing HSCT, 5 out of the 7 AML patients received chemotherapy, with a median of 4 lines of treatment (ranging from 2 to 5). Two out of the seven patients with secondary AML received a azacytidine (vidaza) and venetoclax regimen, and all 4 of the myelofibrosis patients received ruxolitinib. Two patients, 1 from the venetoclax regimen group and 1 from the ruxolitinib group received chemotherapy for a previous malignancy. The donors were sibling, matched-unrelated, and haploidentical for 5, 2, and 4 patients, respectively.

There were several risk factors for developing severe hepatic SOS. These included being over the age of 60, having severe obesity, active illness, undergoing multiple rounds of chemotherapy, and having pre-existing liver disease. The liver-related SOS risk factors included nonalcoholic steatohepatitis cirrhosis without portal hypertension in one patient, splenomegaly in five patients, portal hypertension with esophageal varices in one patient, alcohol abuse in one patient, and baseline liver enzyme elevation in two patients (one with secondary hemochromatosis and the other experiencing severe liver toxicity after previous treatment).

As for the donor and conditioning, two patients had HLA-mismatched and four patients had Haplo-HSCT donors. Four, one, and five patients received MAC, TBI-based, and DAC regimens, respectively.

Five patients received MTX for GVHD prophylaxis. It is worth noting that most patients had multiple factors that put them at risk for SOS, with a median of 5 factors (ranging from 3 to 8).

Out of 11 patients, 6 changed the planned conditioning regimen protocols after LSM indicated a value of >7.0 kPa. The changes included switching from BU to Treosulfan for 3 patients, implementing an alternative protocol to DAC (our routine for myelofibrosis and for haploidentical transplants) for 3 patients, using RIC instead of MAC for 4 patients, and not using MTX for GVHD prophylaxis for 2 patients. One patient received Defibrotide prophylaxis.

Three of 11 patients (27.3%) developed SOS. Two patients developed mild to moderate SOS, while one had severe SOS. The last patient died despite immediate defibrotide treatment.

One of these three patients did not have protocol modified while developing moderate SOS. The LSM levels were 10.1 kPa (IQR, 4.9–11.6) for patients who did not develop SOS, while those who did had LSM levels of 7.4 kPa (IQR, 7.0–22.5). There were no factors that could predict the occurrence of an SOS. However, none of the patients from the high-risk group with an LSM less than 7 kPa developed SOS.

### LSM after HSCT for assessment of SOS

Seventeen patients underwent LSM after their bilirubin levels exceeded 2 mg/dl. The clinical and demographic characteristics of these patients are presented in Table 2. The median age at the time of transplantation was 49 years (IQR, 34–62), and 59% of the patients were male. Ten of these patients were diagnosed with SOS based on EBMT criteria. The LSM was conducted when the bilirubin level increased above 2 mg/dl, regardless of the presence of any other signs of SOS. Out of the 17 patients whose bilirubin level exceeded 2 mg/dl, 7 (41%) had causes of bilirubin elevation other than SOS. Bilirubin elevation in the remaining cases was due to sepsis (*n* = 5) or drug-induced liver injury (*n* = 2). However, due to the small sample size, no statistical differences were found between patients who developed SOS and those who did not. The LSM was performed at a median time of 8.5 days (IQR, 4.25–12.5 days, ranges 1–36 days) after HSCT and 0 days (IQR, 0–1 days, ranges 0–7 days) after bilirubin elevation above 2 mg/dl.

**Table 2:** Baseline demographic and clinical characteristics of patients with bilirubin elevation above 2 mg/dl after allo-HSCT.

Characteristics	All cohort *N* = 17	SOS group *n* = 10	Non-SOS group *n* = 7
Age at allogeneic HSCT (years, median, IQR)	49 (34–62)	53 (32–66)	38 (37–58)
Gender, Male *n* (%)	10 (59)	6 (60)	4 (57)
Obesity, *n* (%)	3 (17.6)	2 (20)	1 (14)
Hepatic comorbidities, *n* (%)			
Baseline bilirubin	0.7 (0.6–0.8)	0.7 (0.6–0.9)	0.7 (0.6–0.9)
Fatty liver disease	1 (7.7)	0	1 (14)
Alcohol misuse	1 (7.7)	0	1 (14)
Portal hypertension	1 (7.7)	1 (10)	0
History of multiple blood transfusion	13 (77)	8 (80)	5 (71)
Ferritin >1000 ng/ml^a^	5	4	1
Diagnosis, *n* (%)			
MDS/AML	7 (41)	4 (40)	3 (43)
ALL	5 (30)	4 (40)	1 (14)
Myelofibrosis	4 (24)	2 (20)	2 (29)
Lymphoma	1 (6)	0	1 (14)
Median Lines of Chemotherapy before HCT	2 (0–6)	3 (0–6)	2 (0–5)
Previous CTX for other malignancy	2 pts	1	1
Active disease at transplant	5 (29)	3 (30)	2 (29)
TBI	4 (24)	3 (30)	1(14)
Conditioning regimen, *n* (%)			
MAC	5	1	4
BUCY	2	1	1
CYTBI	1	0	1
TBF MAC	1	0	1
FLU TREO14	1	0	1
RTC	5	5	0
FLU TREO12	2	2	0
FLUBU4	1	1	0
TBI FLU with PT-CY	2	2	0
RIC	7	4	3
FLU TREO10	2	1	1
FLUBU2	1	1	0
TBF RIC	4	2	2
DAC	5	2	3
GO Tx	1	1	0
Ino Tx	2	2	0

^a^Data of Ferritin level available in 8 patients.

*CTX* chemotherapy, *MF* myelofibrosis, *VV* Vidaza + Venetoclax, *DLBCL* Diffuse large B-cell lymphoma, *TBI* total Body irradiation, *FLU* fludarabin, *RIC* reduced intensity conditioning, *MUD* matched unrelated donor, *TBF* Thiothepa + Busulfan + Fludarabin, *BUCY* Busulfan + Cytoxan, *MUC* myeloablative conditioning, *FC* Fludarabin + Cytoxan.

Patients who developed SOS experienced a higher frequency of abdominal pain, hepatomegaly, ascites, weight gain, and oliguria than those who did not (Table 3). ALT elevation was observed in patients with and without SOS.

**Table 3:** Clinical and laboratory characteristics of patients with and without SOS after HSCT.

Characteristic	SOS *n* = 10	Non-SOS *n* = 7	*P* value
ALT > 1.5 ULN after HSCT, *n* (%)	1 (10)	2 (43)	0.3
Abdominal pain, *n* (%)	9 (90)	1 (14)	0.004
Hepatomegaly, *n* (%)	9 (90)	1 (14)	0.004
Weight gain >5%, *n* (%)	10 (100)	2 (29)	0.003
Ascites, *n* (%)	6 (67)	0	0.01
Oliguria, *n* (%)	6 (60)	0	0.07
Death, *n* (%)	3 (30)	2 (29)	0.9

**Fig. 2:**
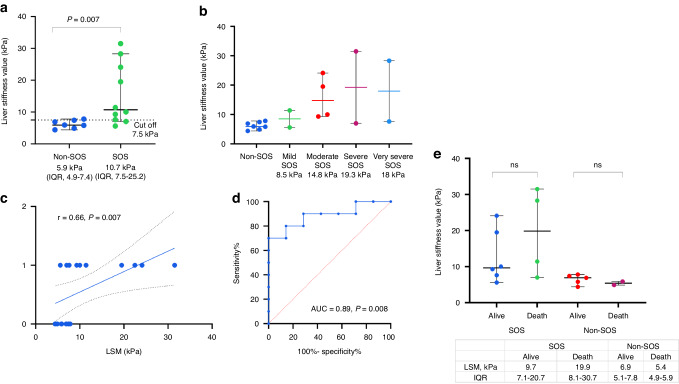
Comparison of liver stiffness measurements (LSM) in patients with and without sinusoidal obstruction syndrome (SOS). **a** Scatter plots displaying the median with interquartile range of LSM values of SOS and non-SOS group, **b** Scatter plots displaying the median with interquartile range of LSM values according to severity of SOS, **c** Regression plot of the Spearman rank correlation between LSM value and SOS diagnosis after HSCT, **d** ROC curve analysis of LSM and prediction of SOS diagnosis after HSCT, **e** Scatter plots displaying the median with interquartile range of LSM values of died and survived patients.

Patients who developed SOS had a significantly higher LSM compared to those who did not. The LSM levels were 5.9 kPa (IQR, 4.9–7.4) for patients without SOS and 10.7 kPa (IQR, 7.5–25.2) for patients with SOS (*p* = 0.007) (Fig. 2a).

We categorized the LSM based on the severity of SOS. As the severity of SOS increased, the median LSM value also increased. For mild, moderate, severe, and very severe cases of SOS, the LSM values were 8.5, 14.75, 19.25, and 17.95 kPa, respectively. However, due to the small sample size, the numbers did not show significant statistical differences (Fig. 2b).

There was a positive correlation between the diagnosis of SOS and LSM levels, with a correlation coefficient of 0.66 (*p* = 0.007) as determined by the Spearman correlation test (Fig. 2c). The LSM can diagnose SOS with an AUC of 0.89 (*p* = 0.008) (Fig. 2d). The cut-off value for SOS diagnosis is 7.5 kPa, with sensitivity 80% and specificity 86%.

During a follow-up of 203 days (IQR, 37–521 days), six patients died. Four patients (40%) died in the SOS group, while two (28.6%) died in the non-SOS group. In the SOS group, only one patient died due to extremely severe SOS with multi-organ failure. Other causes of death included disease progression (two patients), GVHD with severe sepsis, and multi-organ failure (one patient). In the non-SOS group, one patient died due to GVHD with sepsis due to a fungal infection, while the other died due to disease progression.

The median LSM was higher in patients who died compared to those who survived after HSCT in the SOS group (19.9 kPa (IQR, 8.1–30.7) vs. 9.7 kPa (IQR, 7.1–20.7), respectively). However, this difference did not reach statistical significance due to the small sample size. In patients from the non-SOS group, there was no difference in LSM between those who died and those who survived (5.4 kPa (IQR, 4.9–5.9 vs 6.9 kPa (IQR, 5.1–7.8), respectively) (refer to Fig. 2e).

## Discussion

We present the findings from using LSM evaluation both before and after HSCT. We demonstrated that LSM can guide the type of conditioning before transplantation for the high-risk SOS group, leading to possible decrease in the incidence of SOS in this group. We have shown that after a transplant, LSM can be used as a diagnostic tool to differentiate between SOS and other factors that could cause an elevation in bilirubin levels.

After HSCT, harmful events occur, reducing blood flow through the sinusoidal veins [1]. These lead to post-sinusoidal portal hypertension and the onset of SOS. Recently, elastography was used as a non-invasive assessment of portal hypertension by measuring liver stiffness [7].

In our cohort, we observed that the incidence of SOS was 27.3%, which is higher than the reported rate overall [1]. This high incidence of SOS was due to the presence of three or more risk factors for SOS in 81% of the patients. According to a meta-analysis conducted by Coppell et al. in 2010, which included 25,000 pediatric and adult patients, the average incidence of SOS was 13.7% [17]. However, in five studies, the incidence of SOS was greater than 40%, all of which included high-risk patient groups [17].

Assessing liver stiffness before allo-HSCT, we found that none of the patients from the high-risk group and an LSM less than 7 kPa developed SOS. Colecchia and colleagues discovered that LSM increased after HSCT compared to pre-transplant levels [15]. The rise in LSM occurred before the clinical signs of SOS and decreased as patients showed clinical improvement. However, LSM did not significantly change after HSCT in patients who experienced hepatobiliary complications other than SOS. SOS occurred in 5.1% of patients, and those who developed it had higher rates of individual and transplant-specific risk factors than those who did not. Patients who developed SOS also had numerically higher baseline LSM levels [15]. However, Schulz and colleagues reported three cases of fatal SOS in patients who had initially low LSM, but it significantly increased after they developed SOS following transplantation [11]. Further investigation is needed to determine the impact of LSM assessment and how it can contribute to decision-making prior to HSCT and the development of SOS.

We showed that post-allo-HSCT LSM can differentiate between SOS and other causes of bilirubin elevation. We found that an LSM cut-off of 7.5 can predict a diagnosis of SOS with a sensitivity of 80% and a specificity of 86%. Several small studies have described the role of LSM in diagnosing SOS among patients after allo-HSCT [9, 11–15]. Recently, the “ElastoVOD” study conducted in Italy has presented preliminary findings that highlight the effectiveness of LSM in identifying cases of SOS (ClinicalTrial.gov NCT03426358). A study conducted by Debureaux et al. focused on liver stiffness assessment before and after transplantation using TE and 2D-SWE in HSCT patients [9]. The study found a cut-off value of 8.12 kPa using 2D-SWE and 6.87 kPa using TE correlated with an SOS diagnosis 14 days after HSCT [9].

In previous studies, LSM has been examined in patients with varying risk factors for SOS. Our focus was on evaluating high-risk patients and making decisions based on LSM. The additional focus of our study was LSM as an additional criterion to bilirubin elevation for SOS diagnosis. Nonetheless, it is important to acknowledge that our study has several limitations. It is important to acknowledge that the small sample size precludes the extrapolation of our results. We did not perform follow-up liver stiffness assessment in all patients before and after HSCT. However, we can rely only on a single liver stiffness measurement as an additional SOS criterion for bilirubin elevation.

In conclusion, our study suggests that Liver Stiffness Measurement is an important evaluation for candidates for HSCT both before and after transplantation. It may be incorporated as a standard assessment for these patients and can also be used as a criterion for diagnosing SOS. Assessing LSM before allo-HSCT can help determine the best conditioning treatment. However, further investigation is needed to understand the exact impact of LSM on decision-making and the onset of SOS. Future studies are needed to validate the TE method before and after allo-HSCT. Its inclusion in new technological approaches and multifactorial methods will improve the prediction and diagnosis of SOS.

## Data Availability

The data sets generated during the current study are not publicly available for privacy reasons, but are available from the corresponding author on reasonable request.

## References

[1] Mohty M, Malard F, Alaskar AS, Aljurf M, Arat M, Bader P, et al. Diagnosis and severity criteria for sinusoidal obstruction syndrome/veno-occlusive disease in adult patients: a refined classification from the European society for blood and marrow transplantation (EBMT). Bone Marrow Transplant. 2023;58:749–754. 10.1038/s41409-023-01992-8 37095231

[2] Bonifazi F, Barbato F, Ravaioli F, Sessa M, Defrancesco I, Arpinati M, et al. Diagnosis and Treatment of VOD/SOS After Allogeneic Hematopoietic Stem Cell Transplantation. Front Immunol. 2020;11:1–13. 32318059 10.3389/fimmu.2020.00489 PMC7147118

[3] Carreras E, Diaz-Ricart M. The role of the endothelium in the short-term complications of hematopoietic SCT. Bone Marrow Transpl. 2011;46:1495–502. 10.1038/bmt.2011.65 21460864

[4] Bonifazi F, Sica S, Angeletti A, Marktel S, Prete A, Iori AP, et al. Veno-occlusive disease in HSCT patients: Consensus-based recommendations for risk assessment, diagnosis, and management by the GITMO group. Transplantation. 2021;105:686–94. 33273315 10.1097/TP.0000000000003569

[5] Mohty M, Malard F, Abecassis M, Aerts E, Alaskar AS, Aljurf M, et al. Revised diagnosis and severity criteria for sinusoidal obstruction syndrome/veno-occlusive disease in adult patients: A new classification from the European Society for Blood and Marrow Transplantation. Bone Marrow Transpl. 2016;51:906–12. 10.1038/bmt.2016.130 PMC4935979 27183098

[6] Chan SS, Colecchia A, Duarte RF, Bonifazi F, Ravaioli F, Bourhis JH. Imaging in Hepatic Veno-Occlusive Disease/Sinusoidal Obstruction Syndrome. Biol Blood Marrow Transpl. 2020;26:1770–9. 10.1016/j.bbmt.2020.06.016 32593647

[7] de Franchis R, Bosch J, Garcia-Tsao G, Reiberger T, Ripoll C, Abraldes JG, et al. Baveno VII – Renewing consensus in portal hypertension. J Hepatol. 2022;76:959–74. 35120736 10.1016/j.jhep.2021.12.022 PMC11090185

[8] Berzigotti A, Tsochatzis E, Boursier J, Castera L, Cazzagon N, Friedrich-Rust M, et al. EASL Clinical Practice Guidelines on non-invasive tests for evaluation of liver disease severity and prognosis – 2021 update. J Hepatol. 2021;75:659–89. 34166721 10.1016/j.jhep.2021.05.025

[9] Debureaux PE, Bourrier P, Rautou PE, Zagdanski AM, de Boutiny M, Pagliuca S, et al. Elastography improves accuracy of early hepato-biliary complications diagnosis after allogeneic stem cell transplantation. Haematologica. 2021;106:2374–83. 32732366 10.3324/haematol.2019.245407 PMC8409044

[10] Mohty M, Battista ML, Blaise D, Calore E, Cesaro S, Maximova N, et al. A multicentre, multinational, prospective, observational registry study of defibrotide in patients diagnosed with veno-occlusive disease/sinusoidal obstruction syndrome after haematopoietic cell transplantation: an EBMT study. Bone Marrow Transpl. 2021;56:2454–63. 10.1038/s41409-021-01265-2. 10.1038/s41409-021-01265-2 34059801

[11] Schulz M, Vuong LG, Müller HP, Maibier M, Tacke F, Blau IW, et al. Shear wave elastography in the detection of sinusoidal obstruction syndrome in adult patients undergoing allogenic hematopoietic stem cell transplantation. Diagnostics. 2021;11:928. 34064217 10.3390/diagnostics11060928 PMC8224360

[12] Özkan SG, Pata C, Şekuri A, Çınar Y, Özkan HA. Transient elastography of liver: Could it be a guide for diagnosis and management strategy in hepatic veno-occlusive disease (sinusoidal obstruction syndrome)? Transfus Apher Sci. 2022;61:103370. 35101374 10.1016/j.transci.2022.103370

[13] Reddivalla N, Robinson AL, Reid KJ, Radhi MA, Dalal J, Opfer EK, et al. Using liver elastography to diagnose sinusoidal obstruction syndrome in pediatric patients undergoing hematopoetic stem cell transplant. Bone Marrow Transpl. 2020;55:523–30. 10.1038/s41409-017-0064-6. 10.1038/s41409-017-0064-6 29335626

[14] Lazzari L, Marra P, Greco R, Giglio F, Clerici D, Venturini E, et al. Ultrasound elastography techniques for diagnosis and follow-up of hepatic veno-occlusive disease. Bone Marrow Transpl. 2019;54:1145–7. 10.1038/s41409-019-0432-5. 10.1038/s41409-019-0432-5 PMC6760678 30679827

[15] Colecchia A, Ravaioli F, Sessa M, Alemanni VL, Dajti E, Marasco G, et al. Liver Stiffness Measurement Allows Early Diagnosis of Veno-Occlusive Disease/Sinusoidal Obstruction Syndrome in Adult Patients Who Undergo Hematopoietic Stem Cell Transplantation: Results from a Monocentric Prospective Study. Biol Blood Marrow Transpl. 2019;25:995–1003. 10.1016/j.bbmt.2019.01.019. 10.1016/j.bbmt.2019.01.019 30660772

[16] Castera L, Yuen Chan HL, Arrese M, Afdhal N, Bedossa P, Friedrich-Rust M, et al. EASL-ALEH Clinical Practice Guidelines: Non-invasive tests for evaluation of liver disease severity and prognosis. J Hepatol. 2015;63:237–64. 25911335 10.1016/j.jhep.2015.04.006

[17] Coppell JA, Richardson PG, Soiffer R, Martin PL, Kernan NA, Chen A, et al. Hepatic Veno-Occlusive Disease following Stem Cell Transplantation: Incidence, Clinical Course, and Outcome. Biol Blood Marrow Transpl. 2010;16:157–68. 10.1016/j.bbmt.2009.08.024. 10.1016/j.bbmt.2009.08.024 PMC3018714 19766729

